# Retrospective Evaluation of Cryopreserved Human Umbilical Cord Tissue Allografts in the Supplementation of Cartilage Defects Associated with Hip Osteoarthritis

**DOI:** 10.3390/jcm13144040

**Published:** 2024-07-10

**Authors:** Albert Lai, Conrad Tamea, John Shou, Anthony Okafor, Jay Sparks, Renee Dodd, Naomi Lambert, Crislyn Woods, Orion Schulte, Sarah Kovar, Tyler Barrett

**Affiliations:** 1 Desert Physical Medicine and Pain Management, Indio, CA 92201, USA; albertlai@painmedicine.doctor (A.L.); renee@prestigepro.net (R.D.); 2 Orthopedic Associates of Tampa Bay, Tampa, FL 33603, USA; conradtameamd@gmail.com; 3 Department of Pharmacology, Baylor College of Medicine, Houston, TX 77030, USA; drshou@regenativelabs.com; 4 Mathematics & Statistics, University of West Florida, Pensacola, FL 32514, USA; aokafor@uwf.edu (A.O.); jsparks3@uwf.edu (J.S.); 5 Regenative Labs, Pensacola, FL 32501, USA; crislyn@regenativelabs.com (C.W.); orion@regenativelabs.com (O.S.); sarahkovar@regenativelabs.com (S.K.); tyler@regenativelabs.com (T.B.)

**Keywords:** Wharton’s jelly, umbilical cord tissue allografts, cartilage degeneration, hip osteoarthritis

## Abstract

**Background**: Osteoarthritis is a chronic disorder that affects the synovial joints by the progressive loss of articular cartilage. In the hip, the largest weight-bearing joint, the deterioration of articular cartilage and acetabular labrum can cause pain, diminishing the quality of life for patients. This study presents changes in reported pain scales from patients who received Wharton’s jelly applications to cartilage deterioration in the hip from the observational retrospective repository at Regenative Labs. **Methods**: Sixty-nine patients were selected based on inclusion criteria with patient-reported pain scales, including the Numeric Pain Rating Scale and the Western Ontario and McMaster University Osteoarthritis Index, collected at the initial application, 30, and 90-day follow-up visits. Thirteen patients received a second allograft application and had additional follow-up visits at 120 and 180 days. **Results**: Five of the six scales used showed a statistically significant improvement in average scores across the cohort. The greatest improvements were observed in the NPRS with a 31.36% improvement after 90 days and a 44.64% improvement for patients with two applications after 180 days. The minimal clinically important difference (MCID) was also calculated to determine the perceived value of care for each patient with 44.9% of patients exceeding the MCID and 78.3% reporting at least one level of improvement. **Conclusions**: The positive outcomes for the patients in this cohort suggest WJ to be a promising alternative care option for patients with structural tissue degeneration in the hip refractory to the current standard of care.

## 1. Introduction

Osteoarthritis (OA), also known as “degenerative joint disease”, is a chronic disorder that affects the synovial joints through the progressive loss of articular cartilage. The disease typically involves reactive bone formation, osteophyte growth, subchondral cysts, muscle weakness, synovial inflammation, and remodeling. In total, osteoarthritis is a combination of both bone destruction and repair [1,2]. Furthermore, OA is grouped into primary or secondary classifications. Primary, or idiopathic, OA is when no known cause exists, and symptoms occur in multiple joints. Primary OA is generally an exclusion diagnosis and accounts for most hip OA [1]. Secondary OA is a monoarticular condition where symptoms persist in a single joint. Secondary OA develops from a known cause, which is either a defined disorder that affects the joint or trauma that changes the cartilage environment. Secondary OA results from subtle anatomic abnormalities, which makes the hip inclined to mechanical factors that prompt degenerative development [1]. OA often leads to pain, stiffness, swelling, and loss of normal joint function [2]. The hip is commonly affected by OA, being the largest weight-bearing joint in the body. Symptoms of hip OA are gradual and can include unforeseen or progressively worsening pain in the joint. The pain may develop secondary to stiffness in the morning or after a long rest period. As the disease progresses, symptoms may present more frequently regardless of activity level [1]. Degeneration from OA affects all aspects of the joint in its entirety; however, the deterioration of the articular cartilage and acetabular labrum specifically can cause pain from bone-on-bone friction, decreasing the range of motion and, therefore, diminishing the quality of life for patients. While mechanical stress significantly contributes to cartilage degeneration, other risk factors such as age, gender, injury, genetics, and obesity may impact its progression. Biological and biochemical processes within the joint increase the risk of OA, and joint dysplasia is a common condition that is predisposed to hip OA [1]. People who live to the age of 85 have an overall 25% chance of developing symptomatic hip OA [3]. Thus, hip OA is among the most prevalent conditions affecting older populations. For both genders, the risk of OA increases as age increases, but overall, females are at a greater risk of developing the disease [4]. Patient history, physical exam, or imaging techniques help diagnose hip OA. However, a diagnosis can typically be made through patient history and physical examination, excluding radiation exposure to the patient [5]. Once diagnosed, the best practices for patient care are up for debate.

Different management routes fall into educational, behavioral, psychological, physical, and medicinal categories. Strongly recommended therapies excluding medicinal use include exercise, weight loss, self-management programs, and self-efficacy programs [6]. Physicians attempt conservative standard-care options before a patient seeks invasive treatments. Standard patient care includes symptom management with pain treatments like acetaminophen, tramadol, and intraarticular corticosteroid injections [7]. While corticosteroid injections alleviate pain, they risk the progression of cartilage damage and cannot be administered frequently, requiring a three to six-month gap between each injection [8]. Platelet-rich plasma and hyaluronic acid injections can be used as alternatives to corticosteroid injections but are generally either less effective than corticosteroid injections or only effective for a subset of hip OA patients, respectively [8]. Neither option provides a long-term solution for pain relief or directly improves the tissue damage associated with OA. Once a person has reached advanced symptoms and structural damage, they may be a candidate for total joint replacement [9]. Although hip arthroplasty may relieve pain and increase joint stability, several complications are associated with the procedure.

Common complications include infection potentially leading to sepsis, foreign material and space in the wound, hematoma development, nerve injury, thromboembolic disease, femur fracture, asymmetric extremity length, and the loosening of installed components later requiring re-operation, and even pulmonary embolism [10,11]. According to records in the National Hospital Discharge survey, between 1990 and 2004, there were approximately 20 hip revision surgeries for every 100 total hip replacements [11]. With approximately one million arthroplasties performed annually in the US and a thromboembolism incidence rate of 0.6 to 1.5%, a large number of patients are at risk [12]. Total hip arthroplasty procedure costs vary per case but typically fall within the range of $2000 to $13,000 per implant [13]. Patients who undergo an arthroplasty procedure can expect to recover most of their daily functionality within eight months. Still, the outcome is not assessed until a minimum of 2 years after the procedure and may include one or more revisional surgeries [14]. While there are several treatment options to reduce the pain of hip OA, no one protocol addresses the root structural cause of the disorder, indicating the need for further research [5]. Answering the need for new conservative intervention options for cartilage degeneration associated with hip OA, this retrospective study provides statistical evidence of the safety and efficacy of Wharton’s jelly (WJ) applied to the intra-articular space of patients with diminished articular cartilage of the hip.

Human WJ tissue is found within the umbilical cord with the primary function of cushioning and protecting the umbilical vessels from external forces. WJ contains growth factors, collagen (including fiber types I, III, and V), cytokines, proteoglycans, and hyaluronic acid [15]. Human articular cartilage, tendons, and dermal tissues are composed of collagen fibers similar to those found in WJ [16]. Recent studies have shown that when added to standard conservative treatments, WJ effectively improved defects in the articular cartilage of patients with knee osteoarthritis [17]. WJ is among the few immune-privileged connective tissues that, when utilized homologously, may be applied to analogous connective tissue defects that require repair, reconstruction, replacement, or supplementation in the recipient. WJ tissue allografts are procured from the umbilical cords of healthy, live births. The tissue is minimally manipulated and for homologous use only. The FDA guidelines define minimal manipulation as processing that does not alter the original structural characteristics of the tissue. These allografts are not combined with any article except saline and an FDA-approved cryopreservative. Human cells, tissues, and cellular and tissue-based products (HCT/Ps) are regulated to ensure no clinical safety concerns. Staining and imaging assist in confirming the structural integrity of WJ tissue allografts, which are maintained after processing and contain the same collagen matrix found in articular cartilage.

Given the composition of WJ and its successful application in patients with connective tissue defects associated with knee osteoarthritis, we propose that WJ applied directly to the site of tissue defects via syringe in patients with hip osteoarthritis and documented cartilage damage and loss can improve patient tissue defects and quality of life. In this study, we observe data collected over time from patients with hip osteoarthritis who have had WJ allografts applied directly to the deteriorated cartilage, as well as histological evidence, to determine the efficacy and safety of WJ tissue allografts applied in this way.

## 2. Materials and Methods

### 2.1. Study Design

**Figure 1:**
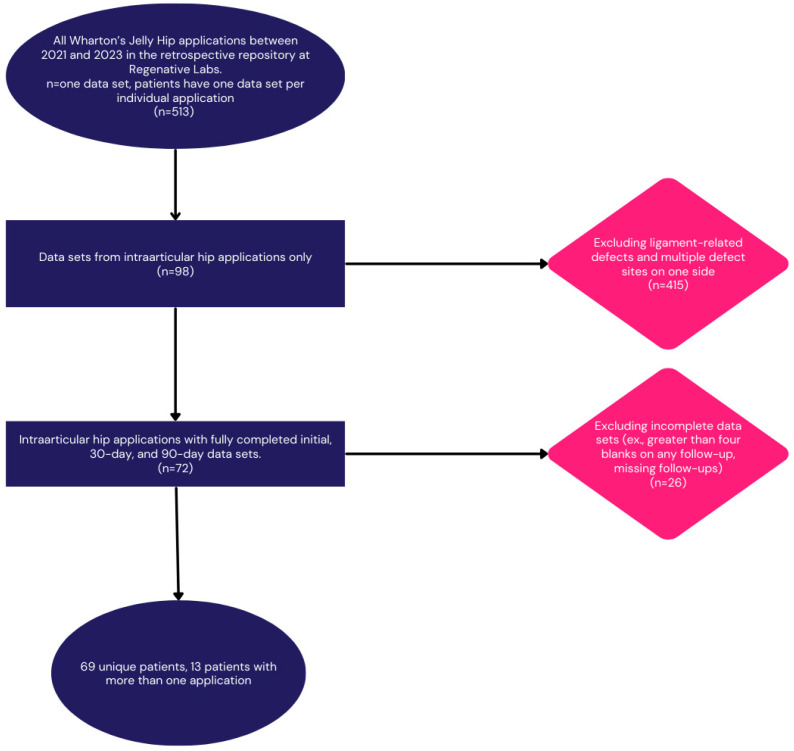
Study design flow chart.

The retrospective repository at Regenative Labs was used to identify the patients analyzed in this paper. The data repository is collected following the guidelines of the Declaration of Helsinki. It has maintained approval from the Institutional Review Board of the Institute of Regenerative and Cellular Medicine (IRCM-2021-311) since May 2021. The repository contains observational data collected from patients who provided informed consent and received one or more applications of either WJ tissue allografts or dehydrated amniotic membrane allografts to any homologous use site. There are over 180 use sites documented to date. The repository has also been used in other publications analyzing different use sites [17,18,19]. Candidates for Wharton’s jelly tissue allografts have objective evidence of structural tissue defects and have failed conservative treatments for at least 90 days. Tissue processing at Regenative Labs, Pensacola, FL, USA, complies with the FDA and American Association of Tissue Banks (AATB) standards, with further manufacturing information available on their website. For this paper, patients with evidence of narrowing joint space and cartilage degeneration in the intraarticular space of the hip joint and treatment-refractory symptoms were selected from the repository. The inclusion and exclusion criteria can be found in the study flowchart below (Figure 1).

### 2.2. Study Population

The study cohort includes 69 patients, 48% female and 52% male. Thirteen of the patients received two applications based on the severity of the defect, five females and eight males. The demographics identify the average age to be 74.5 years old. The average BMI was 28.8 kg/m^2^. The demographics are shown below in Table 1. The patients in this study had exhausted the typical standard-of-care treatment options such as oral non-steroidal medications, topical medications, such as Biofreeze or Salonpas, and physical therapy with rehabilitative instructions. Ultrasound and MRI confirmed the patients had a degeneration of cartilage in the hip. 

### 2.3. Allograft Application

The procedure in this study used a combination of videofluoroscopy and ultrasound. A 3.5-inch spinal needle, under direct ultrasound and video fluoroscopy guidance, was inserted into the hip joint. Applications of 2 mL of the 75 mg/mL WJ tissue allograft (Protext™, Regenerative Labs, 1700 W Main st., Pensacola, FL 32502, USA) and Marcaine followed the verification of proper placement. The WJ tissue allograft tessellates into the damaged cartilage, and Marcaine is a temporary regional anesthetic. A 30-min monitoring period was conducted after each application to ensure no adverse reactions occurred. No complications or adverse reactions were noted in any of the patients. Postoperatively, the patients were to continue with active physical therapy programs, including range of motion and strengthening in the hip exercises. The patients received instructions to avoid strenuous activities, including running, excessive walking, yard work, etc.

### 2.4. Questionnaire Composition

Each patient completed pain scales at the initial application visit, 30 days, and 90 days post-application. Patients who required two applications received their second allograft between 30 and 90 days after the first with subsequent follow-ups at 30 and 90 days after the second application. To assess improvement, patients completed the Numeric Pain Rating Scale (NPRS), Western Ontario and McMaster University Osteoarthritis Index (WOMAC), which included subsections for pain, stiffness, and functionality, and Quality of Life Scale (QOLS) [20,21].

### 2.5. Statistical Analysis

Repeated measures analysis of variance (ANOVA) and Tukey’s tests were performed to assess the difference within groups in outcomes between intervals [22]. This is because the pain scale recorded three different time intervals as a continuous measure, and the values were normally distributed (Shapiro–Wilk test, *p*-value > 0.05). The Wilcoxon rank-sum test was performed on a non-normal distributed population [23]. Statistically significant improvements were observed for applications on either side of the hips after 30 and 90 days in the WOMAC, pain, stiffness, and functionality scales. Analysis of variance (ANOVA) was used to compare the interval changes across all six scales, including any differences observed between gender, age, BMI, and second applications. While ANOVA results represent the significance levels of the intervals, they do not specify which pairs of intervals are different. Accordingly, Tukey’s test was used to identify which specific intervals differ from each other. Additionally, the minimal clinically important difference (MCID) was calculated using the NPRS in the anchor-based method to identify the value by performing a receiver operating characteristic (ROC) curve to determine the perceived value of care to the patients [24,25,26,27]. The MCID was used to identify the minimum, but meaningful differences before and after application, and the anchor-based method was used to identify the value by forming a receiver operating characteristic (ROC) curve.

## 3. Results

**Figure 2:**
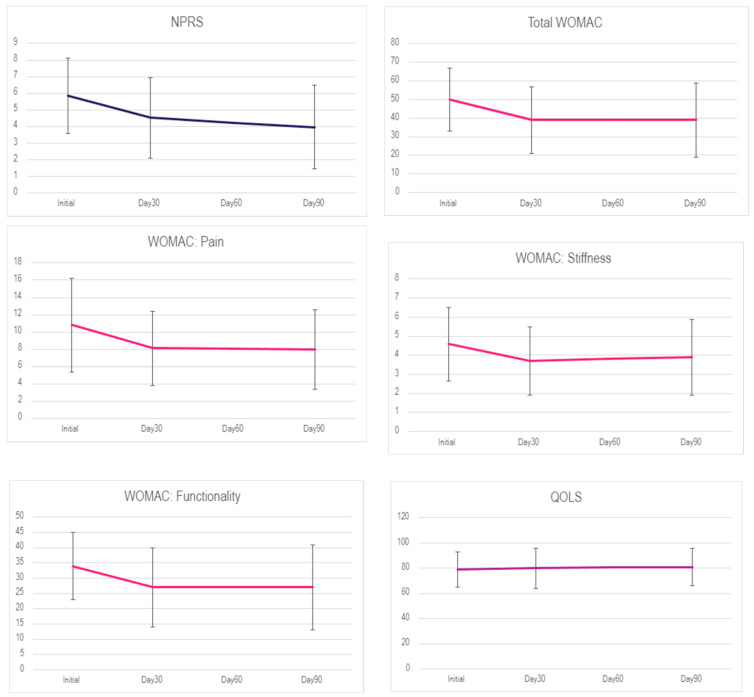
Average score over time from each scale from patients with one application.

When interpreting the results, we must note that for the NPRS and WOMAC scales, a decrease in total score equates to improvement, while for the QOLS, an increase in score equates to improvement. For patients who received one application, the mean pain scores of NPRS and WOMAC declined from the initial application to Day 30 and stayed constant until Day 90. However, the reported QOLS scores did not show any significant change. The mean scores over the time intervals are shown below in Table 2. The subset of patients who received two applications experienced an increase in NPRS and WOMAC scores at 90 days but then a significant decrease in scores after the second application at the 120 and 180-day visits. The mean scores over time in each scale for the 13 patients with two applications are shown below in Table 3 and Figure 2.

The three tests used to analyze data were analysis of variance (ANOVA), Tukey’s test, and Wilcox rank-sum test. Binary outcomes in the data utilized logistic regression. ANOVA was used to compare the interval changes across all scales to determine the statistical significance. ANOVA compares the mean difference between the intervals to see if they are equal. While ANOVA results represent the significance levels of intervals, they do not specify which pairs of intervals are different. Accordingly, Tukey’s test identified which specific intervals differ from each other. ANOVA test results suggested statistically significant changes in the mean pain scores between intervals across five of the six scales: NPRS, WOMAC, pain, stiffness, and functionality. There were no significant differences in QOLS scores for the three intervals. Table 4 shows the actual differences between each interval with lower (LWR) and upper (UPR) bounds at a 95% confidence interval (CI); it also includes the *p*-values from Tukey’s test based on 0.05 to determine significance. As expressed in the *p*-values, there were statistically significant differences in mean pain scores between Initial and Day 30 across scores for NPRS, WOMAC, pain, stiffness, and functionality and between Initial and Day 90 for NPRS, WOMAC, pain, and functionality but not for the interval between Day 30 and Day 90. No statistically significant differences for QOLS existed between the initial application and day 30 or day 90.

Since most pain scales showed remarkable differences between Initial and Day 90, the following analyses determine their mean changes. ANOVA test results showed no differences in age, BMI, and gender in pain scores between Initial and Day 90. The *p*-values for six scales are entered in Table 5 below.

A total of 13 patients received two applications to the same side, and 56 patients received one application. The Wilcoxon rank-sum test compared the differences between single and double applications. When comparing single versus double applications, there were significant differences in the mean changes for NPRS, pain, and QOLS. Thus, single-application patients improved pain scores for total WOMAC, pain, and functionality. Single and double application patients had an average change of 1.76 and 0.00 for NPRS, 3.1 and 0.2 for pain, and −3 and 5 for QOLS with *p*-values less than 0.05. Also, there was no difference in BMI or age between single and double applications. Below are the descriptive data and *p*-values for the two groups.

The average of the changes for single-application patients on NPRS, pain, and QOLS was higher than for double-application patients. This implies that the former had greater improvement than the latter on these scales (*p*-value; 0.21, 0.03, 0.03 for NPRS, pain, and QOLS, respectively). The BMI was averaged at 28 kg/m^2^ for both groups, and mean ages were 74.4 and 75 years for single and double applications, respectively.

To identify the minimum but meaningful differences before and after application, the MCID was used, and the anchor-based method was used to identify the value by performing a receiver operating characteristic (ROC) curve. To measure the improvement over time for the anchor group as a reference, the NPRS score was used from the initial and 90-day visits. After removing the missing scores for NPRS Initial and Day 90, there was a total of 57 patients for the remaining four pain scales and 47 for QOLS. These participants were used to answer the anchor question. The difference in NPRS scores between Initial and Day 90 was calculated. The anchor question can be expressed as, “How different is your pain comparing before and after the application?” After determining the changes, the patients were grouped into four categories by the range of changes in their NPRS scores (Table 6). The grouped answers were “Not better”, “Slightly better”, “Better”, and “Much better”. Table 7 show the descriptive statistics between the answers.

**Figure 3:**
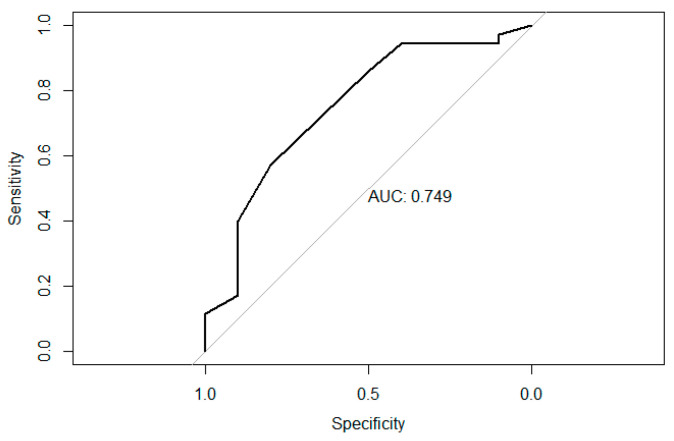
ROC plot for WOMAC-Pain.

The purpose of the grouping is to define the minimally meaningful difference: that is, using the “Not better” and “Slightly better” patient groups as an anchor to determine the MCID. Since these responses are a binary outcome, it is possible to use the ROC curve method to detect the optimal cutoff based on the Area Under the Curve (AUC). The ROC analysis determined the probability of being “Not better” or “Slightly better” and calculated the best cutoff and AUC. The cutoff is commonly based on the highest Youden’s index with the greatest sensitivity and specificity. The closer the AUC is to 1, the less often it misclassifies “Not better”, and the more correctly it identifies “Slightly better”. The useful range of AUC values here was from 0.7 to 0.9 (Figure 3).

The MCID determined the smallest meaningful difference in the WOMAC, pain, stiffness, functionality, and QOLS scores between patients who were “Not better” compared with “Slightly better” according to the anchor questions. Table 8 summarizes these findings.

At least one unit improved: one or more scores improved over Initial.

The MCID values for “Slightly better” patients are 11.14 for total WOMAC, 2.71 for pain, 1.03 for stiffness, 7.40 for functionality, and −5.40. Considering the AUC, the MCID values from the WOMAC and pain scales are more able to discriminate between “Slightly better” or not. About 44.9% exceeded the MCID for WOMAC, and 46.4% exceeded the MCID for pain. The mean difference (MC) and percentage of at least one unit improvement for the entire patient set were computed (Table 9).

Overall, the mean of the five scales improved over time when looking at the MC values for the total population. Between 55.9% and 78.3% of patients improved at least one score after application, depending upon the scale used. While the MCID values for the WOMAC and pain scales were identified, the MCID values for stiffness, functionality, and QOLS were unable to be determined. There were too many variables to establish a baseline anchor group. This may be due to the variance in joints needing additional applications.

## 4. Discussion

This study demonstrated that a 150 mg WJ tissue allograft application presented promising results in supplementing tissue defects associated with hip osteoarthritis in patients who had failed previous standard-of-care treatment options. A statistically significant number of patients reported improved NPRS, WOMAC, and Split WOMAC scores, indicating meaningful improvement in pain and functionality, likely improving a patient’s overall quality of life. Although 78% of patients experienced a positive improvement during the study, there are a few limitations that can be improved in further research. As a retrospective observational study, there is no direct comparison control group. The repository data collection protocol does not have exclusion criteria, and therefore, the sample of patients useable for this study was reduced by our retrospective criteria. Further prospective and randomized control trials can be performed to mitigate these limitations and to reaffirm positive results.

**Figure 4:**
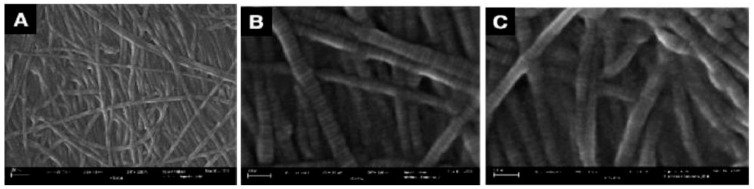
SEM micrographs of unprocessed umbilical cord tissue samples. (**A**) SEM image of cross-linked collagen structures (scale bar: 300 nm). (**B**) SEM image of collagenic structure fibers (scale bar: 100 nm). (**C**) SEM image of collagenic structure fibers (scale bar: 100 nm) [30].

**Figure 5:**
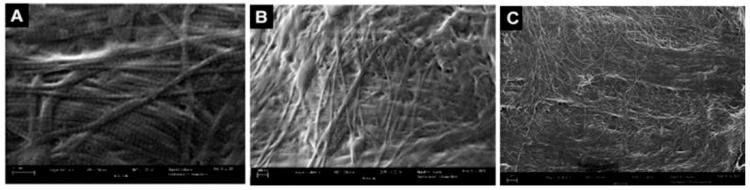
SEM micrographs of umbilical cord tissue post-processing. (**A**) SEM image of preserved cross-linked collagen structures (scale bar: 300 nm). (**B**) SEM image of preserved random directional structural composition of collagen fibers (scale bar: 300 nm). (**C**) SEM image of multidirectional linkage of collagen fibers (scale bar: 1 μm) [30].

**Figure 6:**
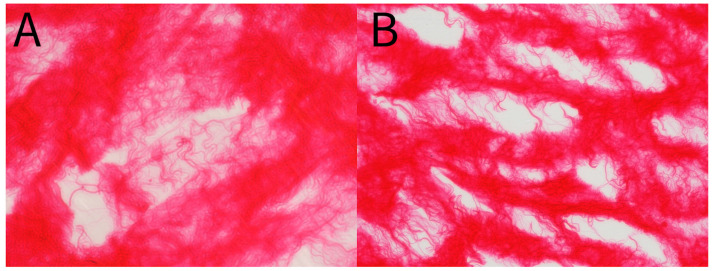
(**A**,**B**) Picro Sirius Red stain of unprocessed umbilical cord tissue sample. (**A**) 40× magnification. (**B**) 20× magnification.

**Figure 7:**
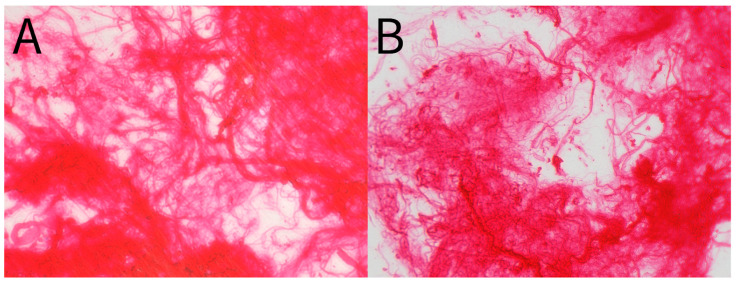
(**A**,**B**) Picro Sirius Red stain of post-processed umbilical cord tissue sample. (**A**) 40× magnification. (**B**) 20× magnification.

**Figure 8:**
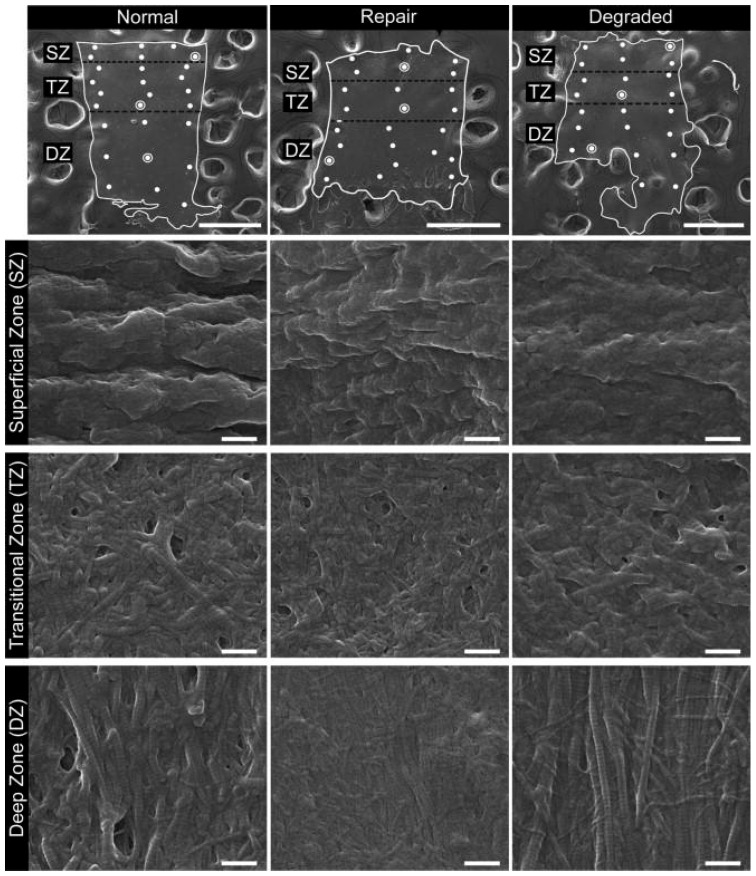
Examples of SEM images for normal, repaired, and degraded cartilages from the superficial, transitional and deep zones. The top row contains the low magnification (80×) SEM images (scale bars are 500 μm) with the non-calcified tissue outlined in white and surrounded by carbon substrate. Zones are identified (SZ, TZ, DZ) as well as the sites where high-magnification images were captured (•). Subsequent rows contain one high-magnification (80,000×) image per zone per cartilage type, and the location from which each image was captured is identified by (⦿) on the corresponding low-magnification image. Scale bars are 500 μm and 500 nm for low and high-magnification images, respectively [31].

Patient-reported scales in this article provide a meaningful frame of reference for the benefits of WJ allografts when applied to cartilaginous defects in the hip. However, with further analysis of the WJ tissue compared to components of hip cartilage, we can confirm the homologous nature of the tissues to support the results documented by patients. The current literature has described WJ as a loose connective tissue that cushions and protects umbilical vessels from tensile stress and strain with high concentrations of collagen types I, III, and V, extracellular matrix proteins, hyaluronic acid, proteoglycans, and growth factors [15,16,28,29]. The tissue allografts observed in this retrospective repository have been imaged with scanning electron microscopy (SEM) and Picro Sirius red stain (PSR) to confirm that the collagen structures maintain their integrity after minimal processing (Figure 4, Figure 5, Figure 6 and Figure 7). The cross-linked collagen fibers are preserved through minimal manipulation practices, visible in Figure 4 and Figure 5, making WJ a viable allograft transplant for collagen-based structural tissues. Picro Sirius red staining under standard light microscopy stains collagen fibers red, while muscle fibers and cytoplasm are stained yellow. Figure 6 displays high collagen concentrations from a cryo-sectioned umbilical cord sample with PSR that is mirrored post-processing (see Figure 7) with the irregular collagen structures maintained. Articular cartilage, which covers the hip socket and the femoral head, and the acetabular labrum are structural tissues designed to cushion and support the joint with similar collagen matrices displayed in SEM in Figure 8. As cartilage degrades, whether from normal aging or accelerated by osteoarthritis, fibers will thin and can change orientation. Corresponding matrices allow WJ tissue allografts to tessellate into damaged cartilage and promote repair.

WJ has demonstrated success in multiple studies involving the integration of regenerative medicine to treatment-refractory use sites. Similar to tissue degeneration associated with hip osteoarthritis, a study was conducted on cartilage defects in the sacroiliac joint. The study included WJ tissue allograft placement in 38 patients with treatment-resistant SI joint pain [19]. Utilizing NPRS scores to rate pain and WOMAC scores to rate function, the study found that 84% of the patients reported lowered NPRS scores from the initial day of application to the 90-day follow-up appointment. Similarly, 76% of patients reported a lowered WOMAC score from the initial application to the 90-day mark [19]. Utilizing the same NPRS and WOMAC scales, a study included 55 participants who presented with the symptomatic degeneration of load-bearing articular cartilage in the knee joint [17]. From the initial application to the 90-day follow-up, the average change in NPRS was two points, producing a statistically significant improvement with a *p*-value less than 0.00001. WOMAC scores had an average change of 2.3 points from the initial day of application to the 90-day follow-up, producing a statistically significant improvement with a *p*-value less than 0.00001. The considerable decrease in patient-reported pain levels in the NPRS and WOMAC scales for both the SI study and the knee osteoarthritis study indicates that the WJ allograft application effectively reduces patient-reported pain in various musculoskeletal locations.

## 5. Conclusions

WJ allografts have been shown to significantly improve self-reported pain scores of patients with degenerated cartilage from hip osteoarthritis. Provided there have been no reported adverse reactions related to the tissue allografts within the span of the retrospective repositories beginning, WJ allografts should be considered as a safe regenerative medicine option for individuals suffering from tissue degeneration refractory to other standard-of-care methods. This study provides the foundation for additional research to clarify the dose, protocol, and durability of WJ allograft applications.

## Figures and Tables

**Table 1:** Patient characteristics.

Characteristic	N	N = 69 ^1^
Age in Years	69	74.5 (6.4) 65.0 91.0
Missing		0
BMI in kg/m^2^	44	28.8 (5.4) 20.7 50.5
Missing		25
Gender	69	
Female		33 (48%)
Male		36 (52%)

^1^ Mean (SD) Minimum Maximum; n (%).

**Table 2:** Sample size and mean (SD) of six scales for each interval for patients with one application.

Interval	N	NPRS ^1^	N	WOMAC ^1^	N	Pain ^1^	N	Stiffness ^1^	N	Functionality ^1^	N	QOLS ^1^
Initial	61	5.85 (2.28)	69	50 (17)	69	10.8 (5.4)	69	4.58 (1.91)	69	34 (11)	63	79 (14)
Day 30	58	4.52 (2.43)	69	39 (18)	69	8.1 (4.3)	69	3.71 (1.78)	69	27 (13)	62	80 (16)
Day 90	59	3.95 (2.51)	69	39 (20)	69	8.0 (4.6)	69	3.89 (1.98)	69	27 (14)	62	81 (15)

^1^ Mean (SD).

**Table 3:** Sample size and mean (SD) of six scales for each interval for patients with two applications.

Interval	N	NPRS ^1^	N	WOMAC ^1^	N	Pain ^1^	N	Stiffness ^1^	N	Functionality ^1^	N	QOLS ^1^
Initial	13	7 (1.63)	13	47.3 (21.9)	13	9.3 (5)	13	4.23 (2.24)	13	33.8 (15.4)	11	82.3 (18.7)
Day 30	11	4.55 (2.11)	13	43.2 (21.4)	13	8.38 (4.89)	13	3.92 (1.85)	13	30.8 (15.2)	12	72.5 (15.4)
Day 120	12	3.58 (1.92)	13	37.6 (19.7)	13	7.7 (4)	13	3.23 (1.83)	13	26.7 (14.5)	9	82.7 (15.9)
Day 180	11	3.9 (2.12)	13	41 (19.5)	13	8 (4.29)	13	3.85 (1.68)	13	29 (13.9)	9	77 (17.77)

^1^ Mean (SD).

**Table 4:** Results of Tukey’s test for each pain scale between intervals.

Scales	Interval	Difference	95% CI (LWR, UPR)	*p*-Value
NPRS	Day 30—Initial	−1.335	−2.38, −0.29	0.008
	Day 90—Initial	−1.903	−2.94, −0.86	0.00
	Day 90—Day 30	−0.568	−1.62, 0.48	0.411
WOMAC	Day 30—Initial	−10.913	−18.31, −3.51	0.002
	Day 90—Initial	−11.159	−18.56, −3.76	0.001
	Day 90—Day 30	−0.246	−7.65, 7.15	0.997
Pain	Day 30—Initial	−2.739	−4.65, −0.83	0.002
	Day 90—Initial	−2.841	−4.75, −0.93	0.002
	Day 90—Day 30	−0.101	−2.01, 1.81	0.991
Stiffness	Day 30—Initial	−0.87	−1.63, −0.11	0.021
	Day 90—Initial	−0.681	−1.44, 0.08	0.09
	Day 90—Day 30	0.188	−0.57, 0.95	0.829
Functionality	Day 30—Initial	−7.304	−12.45, −2.16	0.003
	Day 90—Initial	−7.638	−12.78, −2.49	0.002
	Day 90—Day 30	−0.333	−5.48, 4.81	0.987
QOLS	Day 30—Initial	0.669	−5.82, 7.16	0.968
	Day 90—Initial	1.621	−4.87, 8.11	0.826
	Day 90—Day 30	0.952	−5.56, 7.47	0.936

If *p*-value < 0.05, there is statistical significance.

**Table 5:** Results of Tukey’s test for each pain scale between intervals.

Covariates	NPRS	WOMAC	Pain	Stiffness	Functionality	QOLS
Age	0.149	0.863	0.932	0.321	0.849	0.439
Gender	0.659	0.330	0.781	0.252	0.303	0.762
BMI	0.300	0.262	0.276	0.709	0.340	0.319

For *p*-values > 0.05, there is no difference in changes among age, BMI, or gender.

**Table 6:** Sample size and mean (SD) for five scales in each anchor group.

Scales	N	Not Better, N = 10 ^1^	Slightly Better, N = 35 ^1^	Better, N = 8 ^1^	Much Better, N = 4 ^1^
WOMAC	57	1 (16) −36 27	11 (16) −25 48	11 (12) −2 34	23 (15) 2 39
Pain	57	0.2 (3.3) −6.0 7.0	2.7 (3.3) −6.0 9.0	2.1 (2.3) −2.0 5.0	6.2 (1.5) 5.0 8.0
Stiffness	57	−0.60 (2.17) −4.00 2.00	1.03 (1.84) −4.00 5.00	0.63 (1.19) −1.00 3.00	2.50 (3.51) −1.00 6.00
Functionality	57	1 (12) −26 19	7 (11) −18 35	8 (9) 0 26	14 (14) −5 28
QOLS	47	3 (11) −11 23	−5 (14) −46 29	−2 (3) −6 2	−4 (13) −23 9

^1^ Mean (SD) Minimum Maximum.

**Table 7:** Comparison of changes in pain based on the number of applications.

Characteristic	N	Single Application, ^1^ N = 56	Double Application, ^1^ N = 13	^2^ *p*-Value
NPRS	55	1.76 (2.25) −5.00 7.00	0.00 (1.94) −4.00 2.00	0.021
WOMAC	69	13 (16) −36 56	1 (19) −32 29	0.068
Pain	69	3.1 (4.3) −6.0 23.0	0.2 (4.1) −6.0 7.0	0.033
Stiffness	69	0.80 (2.06) −4.00 6.00	−0.08 (2.02) −4.00 2.00	0.3
Functionality	69	9 (12) −26 43	1 (13) −22 20	0.083
QOLS	57	−3 (13) −23 47	5 (12) −14 29	0.03
Age in Years	69	74.4 (5.8) 65.0 91.0	75.0 (8.7) 65.0 91.0	>0.9
BMI in kg/m^2^	47	28.3 (5.6) 20.7 50.5	28.1 (6.4) 15.7 38.1	0.6
Characteristic	N	Single application, ^1^ N = 56	Double application, ^1^ N = 13	^2^ *p*-value

^1^ Mean (SD) Minimum Maximum/^2^ Wilcoxon rank-sum test. Bold text indicates statistical significance.

**Table 8:** AUC, sensitivity, specificity, and Youden’s index for five scales.

Name	WOMAC	Pain	Stiffness	Functionality	QOLS
AUC	0.71	0.75	0.68	0.69	0.68
Sensitivity	0.83	0.57	0.89	0.83	0.47
Specificity	0.70	0.80	0.50	0.60	0.86
Youden’s Index	0.53	0.37	0.39	0.43	0.32

If the AUC > 0.7, the estimation is useful.

**Table 9:** MCID & percentage exceeding the MCID.

Scales	MCID_AUC_	MC_p_total_	% of Exceed MCID_AUC_	% At Least One Unit Improved
WOMAC	11.14	11.16	44.9	78.3
Pain	2.71	2.84	46.4	76.8
Stiffness	1.03	0.68	26.1	53.6
Functionality	7.40	7.64	43.5	76.8
QOLS	−5.40	−2.66	28.9	55.9

MCID_AUC_: mean changes for “slightly better” anchor group using ROC curve method. MC_p_total_: mean changes for entire patients.

## Data Availability

Data available upon request.
